# De Novo Cancer Incidence after Kidney Transplantation in South Korea from 2002 to 2017

**DOI:** 10.3390/jcm10163530

**Published:** 2021-08-11

**Authors:** Boyeon Kim, Minjin Kang, Yoonjung Kim, Hyung Soon Lee, Banseok Kim, Jung Jun Lee, Yongjung Park, Kyung-A Lee

**Affiliations:** 1 Department of Laboratory Medicine, National Health Insurance Service Ilsan Hospital, Goyang-si 10444, Korea; bkima@yuhs.ac (B.K.); classy81@nhimc.or.kr (B.K.); 2 Department of Laboratory Medicine, Gangnam Severance Hospital, Yonsei University College of Medicine, Seoul 06273, Korea; wsyj1101@yuhs.ac (Y.K.); kal1119@yuhs.ac (K.-A.L.); 3 Research Institute, National Health Insurance Service Ilsan Hospital, Goyang 10444, Korea; kangmj@nhimc.or.kr; 4 Department of Surgery, National Health Insurance Service Ilsan Hospital, Goyang 10444, Korea; soon0925@nhimc.or.kr; 5 Department of Surgery, Gangnam Severance Hospital, Yonsei University College of Medicine, Seoul 06273, Korea

**Keywords:** kidney transplantation, malignancy, cancer risk, population-based study

## Abstract

Advances in patient care and immunosuppressive drugs have improved graft survival, resulting in an increase in kidney transplantation (KT); however, persistent immunosuppression is thought to cause late occurrence of cancer. This population-based study consisted of a total of 14,842 patients whose data from the years 2002 to 2017 were collected from the National Health Information Database in South Korea. Malignancies occurred in 7.6% of the total KT patients. Prostate and thyroid cancers were the most common in males and females, respectively. From the age-adjusted incidence analysis, Kaposi’s sarcoma showed the highest standardized incidence ratio in both male and female patients. According to the linear regression model, cancer incidence in KT recipients under immunosuppressive conditions increased by approximately 0.1% each month. Patients’ age over 39 and the use of prednisolone as an initial steroid regimen were associated with increased risk of cancer development after KT. Our regression and proportional hazards models will help clinicians to predict the approximate cancer incidence risk when monitoring KT recipients. Based on the largest available national database, screening or monitoring methods for cancer detection and prevention can be established for KT patients by considering the factors involved in cancer development.

## 1. Introduction

Kidney transplantation (KT) is the preferred treatment for end-stage renal disease (ESRD) since it leads to substantial improvements in patient survival and quality of life [1]. In the United States, the total number of kidney transplants, including retransplants, remarkably increased from 13,550 in 2001 to 21,247 in 2018 based on the Organ Procurement and Transplantation Network/Scientific Registry of Transplant Recipients (OPTN/SRTR) annual data [2,3]. According to the Korean Network for Organ Sharing (KONOS) statistics, 21,977 KTs were conducted during the 16 years from 2002 to 2017, with a consistent increase in number from 741 in 2002 to 2163 in 2017.

Outcomes after KT have continuously improved, with a current 10-year graft survival of 82% in US kidney recipients [4]. Although the use of immunosuppressive drugs increases graft survival, it also leads to considerable complications such as cardiovascular disease and cancer [5,6,7]. Cancer incidence in KT recipients was reported to be three to five times higher than in the general population [8]. In addition to immunosuppressive drugs, factors such as underlying diseases, previous history of cancer before transplant, age, and sex are also associated with the risk of cancer occurrence in KT recipients [9]. The development of post-transplantation malignancy is one of the primary causes of morbidity and mortality in KT patients [10,11].

The types of cancer reported to occur in KT patients vary across study populations [11]. In Western countries, non-melanoma skin cancer, Kaposi sarcoma, lip cancer, and post-transplant lymphoproliferative disorder (PTLD) are relatively common [10,12,13,14,15]. In Asian countries, renal cancer, gastric cancer, non-Hodgkin lymphoma, and transitional cell carcinoma occur more frequently in KT recipients [16,17,18,19]. Previous studies about Korean KT population are based on either single-center data or nationwide population reported cancer incidence and type of cancer in KT recipients [20,21,22]; however, an up-to-date analysis has to be performed, and the risk factors associated with the development of post-transplantation cancer need to be identified based on the most current nationwide population data.

This study was based on the National Health Information Database (NHID) containing all records of healthcare utilization by KT recipients. In this study, we investigated the types and risk factors of post-KT de novo cancer in a population cohort of the NHID in South Korea from 2002 to 2017.

## 2. Materials and Methods

### 2.1. Study Subjects and Data

The Korean National Health Insurance Service (NHIS) operates the NHID to provide national health data. Data from 21,191 single-organ KTs performed in South Korea during the 16 years from 2002 to 2017 were reviewed. If the same patient underwent two or more KTs, only the data before re-transplantation were included. Consequently, the data from a total of 20,750 patients in the NHID were analyzed.

Among those patients, 2219 were excluded due to a history of KT before 2002. These patients were defined as having either a history of continuous immunosuppressant medication or a diagnosis with the Korean Standard Classification of Disease (KCD)/International Statistical Classification of Diseases and Related Health Problems 10th Revision (ICD-10) code Z94.0 ‘Kidney transplant status’ prior to the KT performed during the study period. In addition, 200 patients with unclear history of immunosuppressants during KT hospitalization were excluded. Of the remaining 18,331 patients who underwent first KT, only 17,089 patients who initially received a calcineurin inhibitor, either cyclosporine or tacrolimus, as an immunosuppressive medication during KT hospitalization were included to this study to analyze cancer-related factors. In addition, 2247 patients were excluded due to a previous history of cancer diagnosis before KT; thus, a total of 14,842 patients were finally evaluated to identify the incidence of various de novo cancers. We divided male and female patients in age groups with 5-year intervals in order to make a comparison with the annual report of the Korea Central Cancer Registry (KCCR). In the KCCR report, patient groups are divided by age with 5-year intervals.

We reviewed incidence and timing of the clinical outcomes of KT rejection (ICD-10 codes T86 or T86.1), cancer diagnosis (ICD-10 codes C00 to C97), and death during the post-KT follow-up period. Due to the nature of the NHID, specifying the exact time of rejection was not possible. Therefore, acute kidney allograft rejection was defined as cases for which a diagnosis of graft rejection was recorded during the hospitalization period for KT. Additionally, incidence of cancer was defined as cases with the same ICD-10 code registered as the main diagnosis at least twice after KT in order to exclude cases for which cancer diagnosis was accidentally registered. In South Korea, all patients with cancer diagnosis are registered as ‘cancer patients’ at the National Cancer Information Center (NCIC) via the NHID after undergoing the necessary diagnostic processes including pathological confirmation, and the patients’ sharing rate of the medical cost is reduced for all ‘cancer patients’ after registration. Therefore, incorrect registration as a ‘cancer patient’ can be ruled out through the NCIC registration processes. This study was approved by the Institutional Review Board of the National Health Insurance Service Ilsan Hospital (IRB no. NHIMC-2019-01-001).

### 2.2. Statistical Analyses

Only the data up to the time of death were included in the analysis for de novo cancer incidence. Cancer incidence during the follow-up period after KT between 2002 and 2017 was compared to the data from the KCCR [23], which is operated by the NCIC and includes the age-adjusted cancer incidence per 100,000 person-years. The standardized incidence ratio (SIR) was calculated as follows:

$$\mathrm{Standardized}\;\mathrm{incidence}\;\mathrm{ratio}=\frac{\mathrm{Observed}\;\mathrm{cancer}\;\mathrm{incidence}}{\mathrm{Age}-\mathrm{adjusted}\;\mathrm{expected}\;\mathrm{cancer}\;\mathrm{incidence}}$$

The observed cancer incidence was calculated by dividing the observed cancer cases by death-censored person-years during the study period. The age-adjusted expected cancer incidence was calculated by adjusting the 2017 annual cancer incidence of the KCCR according to the age-groups with 5-year intervals with the person-years according to the same age-group of our data. All analyses were performed separately by sex. To analyze the cumulative cancer incidence, Kaplan–Meier survival analysis was applied, and then linear regression was used to determine the equation for predicting cancer incidence according to follow-up after KT. Cox proportional hazards regression as a multivariate analysis was performed to investigate the hazard ratio (HR) of each variable by mutually correcting the effects of each factor on cancer occurrence. For all statistical analyses, *p* < 0.05 was considered statistically significant. All statistical analyses were performed using SAS software, version 7.15 (SAS Institute Inc., Cary, NC, USA) and RStudio software, version 1.1.463 (RStudio Inc., Boston, MA, USA).

## 3. Results

### 3.1. Patient Characteristics

The number of KT patients per year constantly increased from 452 in 2002 to 1454 in 2017. Among the total 14,842 patients included, 8729 (58.8%) were male, the mean age at the time of KT was 46.0 ± 12.4 years, and those aged between 50 and 59 accounted for the largest portion (30.3%), followed by those in their 40s (27.4%) and 30s (19.3%) (Supplementary Tables S1 and S2). A median follow-up period was 66 months (mean = 73.1 months; min to max = 0 to 194 months; 1st to 3rd quartiles = 28 to 109 months).

### 3.2. Cancer Incidence

The cancer incidence and number of cancer patients during the entire follow-up period are shown in Supplementary Table S3. During the study period, 1050 (7.6%) de novo cancer cases among 13,912 (death-censored) patients developed, including 605 cases in males and 445 cases in females. Compared to the age-unadjusted statistics from the NCIC [24], the cancer incidence in KT recipients ranged from 2.2 to 3.0 times higher than that of the South Korean population from 2010 to 2016 (Supplementary Table S3).

The age-adjusted SIRs of all de novo primary cancers according to the age groups during the 16-year follow-up period are shown in Table 1. The overall SIRs of KT patients decreased as age increased. In male patients, the SIRs for the age groups 5–9, 10–14, 15–19, 20–24 showed statistically significant high values over 25.0. In female patients, the same age groups showed a significantly high SIR over 15.0. Overall, the age-adjusted cancer risk of KT patients was higher than that of the general population.

The age-adjusted SIRs by de novo primary cancer types after KT during 16 years are summarized in Table 2. The SIR for all cancers was 2.5 [95% confidence interval (CI) = 2.2 to 2.8] in males and 2.6 (95% CI = 2.3 to 2.9) in females. The highest SIR was shown for Kaposi’s sarcoma in both males (184.5, 95% CI = 23.8 to 1428.9) and females (341.3, 95% CI = 38.1 to 3053.0), with statistical significance (*p*-value < 0.0001). In males, prostate cancer (*n* = 104, 17.2%), renal cancer (*n* = 97, 16.0%), liver cancer (*n* = 83, 13.7%), and non-Hodgkin lymphoma (*n* = 64, 10.6%) were the most commonly developed cancers. In females, thyroid cancer (*n* = 105, 23.6%), breast cancer (*n* = 61, 13.7%), liver cancer (*n* = 43, 9.7%), and non-Hodgkin lymphoma (*n* = 37, 8.3%) were the most commonly developed cancers. In males, the SIRs for prostate, renal, liver cancers, and non-Hodgkin’s lymphoma were, respectively, as follows, with statistical significance: 5.2, 95% CI = 3.6 to 7.4, *p*-value < 0.0001; 11.3, 95% CI = 6.8 to 18.7, *p*-value < 0.0001; 3.2, 95% CI = 2.3 to 4.5, *p*-value < 0.0001; 12.2, 95% CI = 6.4 to 23.1, *p*-value < 0.0001. In females, the SIRs of thyroid, breast, liver cancers, and non-Hodgkin lymphoma were, respectively, as follows, with statistical significance: 2.6, 95% CI = 2.0 to 3.4, *p*-value < 0.0001; 1.3, 95% CI = 1.0 to 1.8, *p*-value = 0.0387; 9.4, 95% CI = 5.1 to 17.5, *p*-value < 0.0001; 13.2, 95% CI = 6.1 to 28.2, *p*-value < 0.0001.

**Figure 1:**
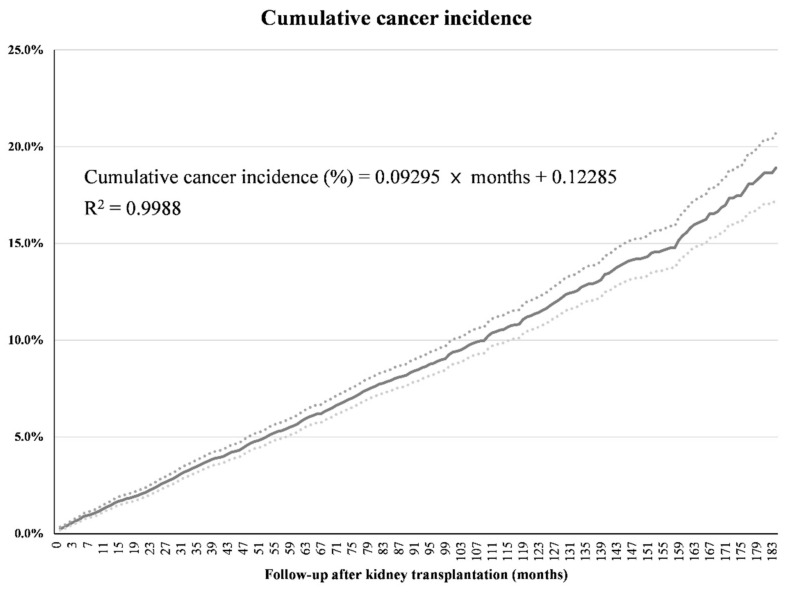
Cumulative cancer incidence rate using Kaplan–Meier analysis during the follow-up period after kidney transplantation. The dashed lines indicate the 95% confidence interval (CI) of the cumulative cancer incidence according to the length of the follow-up (in months). The equation was derived using data from up to 157 months of follow-up; the 95% CI of the slope and y-intercept are 0.09245 to 0.09345 and 0.07737 to 0.16834, respectively.

The incidence of cancer according to the length of follow-up is shown in Table 3. The cumulative cancer incidence at 12, 24, 60, 120, and 180 months after KT was 1.4%, 2.3%, 5.6%, 11.2%, and 18.4%, respectively. Until 157 months after KT, the cumulative cancer incidence rate increased linearly with the length of the follow-up period (Figure 1). The following equation was determined from our data: Cumulative cancer incidence (%) = 0.09295 × months after KT + 0.12285, which had an R2 of 0.9988 and 95% CI for the slope and y-intercept of 0.09245 to 0.09345 and 0.07737 to 0.16834, respectively.

Supplementary Table S4 demonstrates the types of post-kidney transplantation de novo cancers, including different types occurring in the same patient. A total of 13,912 (8142 male patients and 5770 female patients) death-censored KT recipients were analyzed. The most common cancer type occurred in the thyroid, accounting for a total of 154 cases, with an incidence of 1.1%. In addition, a total of 130 kidney cancers corresponded to an incidence of 0.9%. Non-Hodgkin lymphoma was diagnosed in 99 KT recipients, with an incidence of 0.7%. Additionally, a total of 15 cases of Kaposi’s sarcoma corresponded to an incidence of 0.1%.

The results of the analysis on cancer incidence-related risk factors using the Cox proportional hazards regression model are shown in Table 4. Compared to patients younger than 20, patients older than 39 had a higher cancer risk. Patients treated with (methyl)prednisolone as the initial steroid regimen compared to deflazacort had a higher risk of being diagnosed with cancer. Among the analyzed factors, sex, income level, year and organization of surgery, use of anti-thymocyte globulin (ATG), basiliximab, or rituximab, type of initial calcineurin inhibitor, use of mycophenolate mofetil, and the history of acute rejection did not significantly affect cancer development.

## 4. Discussion

Cancer was reported to be the most common cause of death after 20 years in KT recipients with functioning grafts [10]. A national survey in Japan showed that PTLD, kidney, stomach, colon, and lung cancer were most common in solid organ transplantation recipients [25]. In addition, single-center data from China reported that urothelial transitional cell carcinoma, hepatocellular carcinoma, gastrointestinal cancer, and renal cell carcinoma were most common [18]. According to the Korean NCIC data for 2017, the most common type of cancer in the general Korean population was stomach cancer, followed by colon, lung, and thyroid cancers [24].

In this study, 16 years (2002 to 2017) of information in the NHID from South Korea were utilized, and age- and gender-adjusted data were analyzed. As a result, thyroid cancer was the most common in female KT recipients, followed by breast and liver cancers, non-Hodgkin lymphoma, and stomach and kidney cancers. In male KT patients, prostate cancer was the most common, followed by kidney and liver cancers, non-Hodgkin lymphoma, and stomach cancer (Table 2). Considering the SIR, the incidence of Kaposi’s sarcoma increased the most in both male and female KT patients compared to the general population. In male, the risk of developing non-Hodgkin lymphoma and kidney cancer was higher compared to the general population. Also, bladder cancer, non-Hodgkin lymphoma, and kidney cancer showed higher SIRs in females. Considering that the types of cancer occurring in the general population and KT recipients were dissimilar, different approaches from typical cancer screening methods are recommended to monitor cancer development in KT recipients.

The high incidence of thyroid cancer in females (Table 2) is unique among the Asian countries previously studied. The overdiagnosis of thyroid cancer via overutilization of ultrasonography in South Korea may be associated with this high incidence [26]. However, some studies found that the incidence of thyroid cancer was elevated in KT recipients [27,28]. Even in patients with ESRD, thyroid diseases such as hypothyroidism or thyroid nodules were commonly noted due to altered hormone excretion and transport [29,30]. In addition, our study showed that the SIR for thyroid cancer is 2.6 in KT patients, with statistical significance (Table 2). It should also be noted that the incidence of kidney, liver, and stomach cancers in this study showed a similar trend to those reported in other Asian countries. Therefore, further cohort studies may be helpful to determine the exact cause of the high incidence of thyroid cancer in Korean KT recipients.

Although there are still opposing opinions [31,32], the highest incidence of prostate cancer in males was observed in our study (Table 2). According to Sherer et al., the use of older immunosuppressants such as cyclosporine, azathioprine, and tacrolimus may increase the risk of developing prostate cancer compared to the use of newer agents such as the mammalian target of rapamycin (mTOR) inhibitor [31]. In this study, we could not find the increased risk of cancer development according to the types of initial calcineurin inhibitors (Table 4). However, the SIR for prostate cancer was 5.2 in our data, which still shows that male KT patients have a higher risk of prostate cancer development compared to the general population (Table 2). Therefore, careful prostate monitoring for male KT patients would be helpful.

In the Cox proportional hazards regression model, patients over 39 years of age showed a significantly higher HR for cancer than younger patients. However, the cancer incidence increases with age also in the general population. Nevertheless, most age groups of our KT recipients showed increased SIRs compared to the general population (Table 1). This implies that the increased incidence of de novo cancers in KT recipients would be affected by factors related to the post-KT status as well as age. Furthermore, the type of initial immunosuppressive drugs used for KT patients did not significantly affect the risk of developing cancer. It has been known that calcineurin inhibitors, such as tacrolimus and cyclosporine, can increase the production of transforming growth factor β1 in tumor cells [33,34]. Cyclosporine also increases the expression of vascular endothelial growth factor, which can lead to angiogenesis, supporting tumor growth [35]. In this study, cancer risk was not significantly different between patients treated with tacrolimus versus cyclosporine. However, we could not identify the effect of calcineurin inhibitors as a group on cancer development because all KT recipients in our study had received one of the calcineurin inhibitors. Furthermore, the use of mycophenolate mofetil did not significantly alter the cancer risk after KT. Meanwhile, the cumulative dose of steroids significantly increased cancer occurrence in a previous study [36]; however, the type of initial steroid regimen did not significantly affect cancer risk in our study, except for (methyl)prednisolone. Moreover, as many studies previously determined [37,38], the perioperative use of ATG was not associated with cancer risk in this study.

In addition, we were able to derive an equation to estimate the cumulative cancer incidence according to the length of the follow-up period, owing to the large dataset utilized. According to our model, the cumulative cancer incidence in KT recipients under immunosuppressive conditions increases by approximately 0.1% each month. This may help clinicians to predict the approximate cancer incidence risk when monitoring KT recipients. It is expected that this trend in cumulative cancer incidence can be referenced in determining the frequency of cancer screening for long-term follow-up patients. Furthermore, clinicians need a better prevention to lessen the linear occurrence or risk of an increase in cancer incidence.

Currently, a similar study about the incidence of malignancy and related mortality in 9,915 KT recipients using the database of the Health Insurance Review and Assessment Service (HIRA) from 2003 to 2016 has been published [22]. Compared to that study, we used NHID, a more comprehensive database which includes the HIRA database. Therefore, a total of 14,842 KT recipients were included in our study, although ours and that study inspected a similar time period. The incidence of cancer was similar based on the result of 6.0% in that study and of 7.5% in our study. We categorized the cancer types based on the ICD-10 code classification for malignancy from the 2017 KCCR annual data, but the definition of cancer types according to the ICD-10 code is not evident in the study of Jeong et al. Therefore, the incidence according to the types of cancer in their study differs from our results. Furthermore, we calculated SIRs by adjusting age every 5 years using the open access annual report of cancer statistics in Korea in 2017 published by the KCCR [23]. The annual cancer incidence from 2012 to 2017 in Korea remained around 450 cases per 100,000 persons [24]; thus, we used the most up-to-date and detailed annual report (2017) from the KCCR for calculating SIRs. For these reasons, the SIR for each cancer type in our study would be reliable, owing to the largest cohort size among studies using Korean national data, objective classification of cancer types, and application of an age-adjusted database.

Because this study was based only on health insurance claims data, it has several limitations. Other clinical factors, such as laboratory, imaging, or pathology reports, regimen and duration of immunosuppressive therapy, time on dialysis, patient compliance, and standards of clinical practices could not be taken into consideration. Also, the methods used for screening cancer development as well as social factors such as smoking, alcohol use, and family history could not be reported. Furthermore, there is a recent report that a pretransplant malignancy was associated with the graft survival [39]; however, we analyzed the data to determine only the de novo cancer incidence after KT, so we were unable to examine the association between cancer incidence and graft outcome. In addition, thanks to the European Senior Program in Europe, a systematic research about factors related to the prognosis of KT in elderly people was recently reported [40]. However, there is no such program in South Korea for the elderly; therefore, the association between cancer risk and the possibility of kidney transplants being carried out differently depending on age could not be evaluated. Nevertheless, to the best of our knowledge, our data represent the largest KT recipient population and the longest follow-up period among Asian studies. In addition, only data from the first KT (i.e., data collected before re-transplantation) and from patients without any previous cancer history were included. Therefore, this study is representative of the incidence of cancers in KT recipients in South Korea. Although 16 years of data were used in this study, additional prospective cohort studies with longer periods of time should be conducted to provide additional insights regarding cancer development in KT recipients and to complement the limitations of this research.

In conclusion, the incidence and risk factors of cancer in a KT-recipient population using a large dataset from 2002 to 2017 were analyzed in this study. The age-adjusted SIRs of all de novo primary cancers and each cancer type after KT, as well as an equation to help in the prediction of cancer incidence in KT patients based on the length of time after transplantation in our results would be helpful. Furthermore, this study confirmed that the common types of cancer in South Korea were different from those in other countries. Therefore, geographical and racial variations should be considered when KT recipients are screened or monitored for early detection of cancer.

## Figures and Tables

**Table 1:** Age-adjusted standardized incidence ratio of all de novo primary cancers after kidney transplantation during 16 years.

Age Group (Years)	Male							Female						
	KCCR Incidence ^1^	Person-Years (*N*)	Observed (*N*)	Expected (*N*)	SIR	95% CI	*p*-Value	KCCR Incidence ^1^	Person-Years (*N*)	Observed (*N*)	Expected (*N*)	SIR	95% CI	*p*-Value
0–4	20	12	0	0.0	-	-	-	17	13	0	0.0	-	-	
5–9	11	96	1	0.0	94.7	(12.4–726.3)	<0.0001	11	79	1	0.0	115.1	(15.0–880.8)	<0.0001
10–14	13	277	2	0.0	55.5	(12.6–245.0)	<0.0001	14	184	3	0.0	116.5	(33.8–401.9)	<0.0001
15–19	20	534	3	0.1	28.1	(8.4–94.3)	<0.0001	20	316	1	0.1	15.8	(2.1–117.5)	0.0035
20–24	24	958	6	0.2	26.1	(10.7–63.7)	<0.0001	49	569	7	0.3	25.1	(11.4–55.2)	<0.0001
25–29	44	1627	3	0.7	4.2	(1.3–13.5)	0.0081	109	1153	10	1.3	8.0	(4.2–15.2)	<0.0001
30–34	81	3263	10	2.7	3.8	(2.0–7.3)	<0.0001	188	2430	10	4.6	2.2	(1.2–4.1)	0.0078
35–39	113	5010	29	5.7	5.1	(3.4–7.7)	<0.0001	289	3730	34	10.8	3.2	(2.2–4.5)	<0.0001
40–44	177	6077	48	10.8	4.5	(3.3–6.1)	<0.0001	424	4294	57	18.2	3.1	(2.4–4.1)	<0.0001
45–49	245	6712	61	16.4	3.7	(2.8–4.9)	<0.0001	502	5012	64	25.2	2.5	(2.0–3.3)	<0.0001
50–54	427	7716	91	33.0	2.8	(2.2–3.5)	<0.0001	579	5634	71	32.6	2.2	(1.7–2.8)	<0.0001
55–59	701	6541	119	45.8	2.6	(2.1–3.2)	<0.0001	606	5434	73	32.9	2.2	(1.7–2.8)	<0.0001
60–64	1075	5060	102	54.4	1.9	(1.5–2.3)	<0.0001	708	3787	64	26.8	2.4	(1.9–3.1)	<0.0001
65–69	1639	2685	81	44.0	1.8	(1.5–2.3)	<0.0001	780	1892	38	14.8	2.6	(1.9–3.6)	<0.0001
70–74	2192	909	40	19.9	2.0	(1.5–2.7)	<0.0001	951	551	11	5.2	2.1	(1.2–3.8)	0.0068
75–79	2675	190	7	5.1	1.4	(0.7–2.9)	0.1944	1141	79	0	0.9	-	-	
≥80	3051	32	2	1.0	2.1	(0.5–7.8)	0.1475	1295	8	1	0.1	9.7	(1.5–60.4)	0.0078

Abbreviation: KCCR, Korea Central Cancer Registry; SIR, standardized incidence ratio; CI, confidence interval. ^1^ 2017 annual cancer incidence per 100,000 persons according to age groups.

**Table 2:** Age-adjusted standardized incidence ratio by de novo primary cancer types after kidney transplantation during 16 years.

Sex	Type/Site of Cancer	ICD-10	KCCR Incidence ^1^	Observed (*N*)	Expected (*N*)	SIR	95% CI	*p*-Value
Male								
	All primary cancers	C00–C96	478.1	605	239.8	2.5	(2.2–2.8)	<0.0001
	Prostate	C61	50.0	104	20.0	5.2	(3.6–7.4)	<0.0001
	Kidney	C64	14.1	97	8.7	11.3	(6.8–18.7)	<0.0001
	Liver	C22	45.0	83	26.2	3.2	(2.3–4.5)	<0.0001
	Non-Hodgkin lymphoma	C82–C86, C96	10.6	64	5.3	12.2	(6.4–23.1)	<0.0001
	Stomach	C16	77.9	62	43.1	1.4	(1.1–2.0)	0.0129
	Thyroid	C73	23.6	49	15.6	3.1	(2.0–4.8)	<0.0001
	Lung	C34	72.9	39	30.2	1.3	(0.9–1.9)	0.1003
	Colon	C18	37.1	34	18.9	1.8	(1.1–2.8)	0.0066
	Skin, except malignant melanoma	C44	9.8	34	4.2	7.9	(3.8–16.5)	<0.0001
	Pancreas	C25	14.6	26	7.1	3.6	(1.9–6.9)	<0.0001
	Bladder	C67	13.8	21	5.7	3.7	(1.8–7.5)	0.0002
	Rectum	C19, C20	28.0	19	15.7	1.2	(0.7–2.1)	0.2567
	Kaposi’s sarcoma	C46	0.2	11	0.1	184.5	(23.8–1428.9)	<0.0001
	Gallbladder	C23, C24	13.9	8	5.5	1.4	(0.6–3.4)	0.2316
	Myeloid leukemia	C92–C94	5.2	7	2.6	2.5	(0.8–7.3)	0.0539
Female								
	All primary cancers	C00–C96	428.6	445	173.7	2.6	(2.3–2.9)	<0.0001
	Thyroid	C73	78.5	105	40.0	2.6	(2.0–3.4)	<0.0001
	Breast	C50	86.9	61	46.4	1.3	(1.0–1.8)	0.0387
	Liver	C22	15.2	43	4.4	9.4	(5.1–17.5)	<0.0001
	Non-Hodgkin lymphoma	C82–C86, C96	8.0	37	2.7	13.2	(6.1–28.2)	<0.0001
	Stomach	C16	38.1	33	13.3	2.5	(1.6–3.9)	<0.0001
	Kidney	C64	6.6	33	2.7	11.7	(5.4–25.4)	<0.0001
	Colon	C18	28.2	30	8.6	3.6	(2.1–6.1)	<0.0001
	Ovary	C56	10.5	25	4.8	5.1	(2.6–9.8)	<0.0001
	Cervix uteri	C53	13.5	19	6.3	3.0	(1.6–5.7)	0.0004
	Rectum	C19, C20	16.5	17	5.7	3.0	(1.5–6.0)	0.0007
	Skin, except malignant melanoma	C44	12.6	16	2.3	6.5	(2.7–15.8)	<0.0001
	Pancreas	C25	12.9	14	3.1	4.4	(1.9–10.2)	0.0003
	Bladder	C67	3.3	14	0.8	19.9	(4.5–87.6)	<0.0001
	Lung	C34	32.5	13	9.8	1.3	(0.7–2.6)	0.2038
	Kaposi’s sarcoma ^2^	C46	0.1	4	0.0	341.3	(38.1–3053.0)	<0.0001

Abbreviations: KCCR, Korea Central Cancer Registry; SIR, standardized incidence ratio; CI, confidence interval. ^1^ 2017 annual cancer incidence per 100,000 persons, age-unadjusted; ^2^ Not listed in order of incidence.

**Table 3:** Cumulative incidence of all de novo primary cancers according to length of the follow-up after kidney transplantation by Kaplan–Meier analysis.

Follow-Up Period (Months)	Cumulative Cancer Incidence (%)	95% CI (%)
0	0.3	0.2–0.4
1	0.3	0.3–0.4
3	0.6	0.5–0.7
6	0.9	0.8–1.1
9	1.1	0.9–1.3
12	1.4	1.2–1.6
18	1.9	1.6–2.1
24	2.3	2.1–2.6
30	3.0	2.9–3.3
36	3.6	3.3–3.9
42	4.0	3.7–4.4
48	4.6	4.2–5.0
54	5.1	4.7–5.6
60	5.6	5.2–6.0
72	6.7	6.3–7.2
84	7.9	7.3–8.4
96	8.8	8.2–9.4
108	10.0	9.3–10.7
120	11.2	10.5–12.0
132	12.5	11.7–13.4
144	13.9	12.9–14.9
156	14.7	13.7–15.8
169	16.6	15.4–18.0
180	18.4	16.9–20.1

Abbreviation: CI, confidence interval.

**Table 4:** Risk factors and multivariate hazard ratios by the Cox proportional hazards model for occurrence of any de novo primary cancer in 14,842 patients after kidney transplantation.

Variables	No. of Patients (%)	HR	95% CI	*p*-Value
Male	8729 (58.8%)	1.0	(0.9–1.1)	0.5887
Age at KT				
<20	393 (2.6%)	1.0		
20–39	3948 (26.6%)	1.1	(0.7–1.8)	0.6438
40–59	8565 (57.7%)	**2.3**	**(1.4–3.6)**	**0.0007**
≥60	1936 (13.0%)	**4.0**	**(2.4–6.5)**	**<0.0001**
Income, upper 50th percentile	860 (5.8%)	1.0	(0.8–1.3)	0.7321
Date of surgery				
2002–2005	1899 (12.8%)	1.0		
2006–2009	3110 (21.0%)	1.1	(0.9–1.3)	0.6076
2010–2013	4487 (30.2%)	1.1	(0.9–1.4)	0.3209
2014–2017	5346 (36.0%)	1.1	(0.9–1.5)	0.3765
Institution type				
General hospital	2178 (14.7%)	1.0		
Tertiary hospital ^1^	12,664 (85.3%)	1.0	(0.9–1.3)	0.6232
Perioperative use of				
Anti-thymocyte globulin	1836 (12.4%)	1.0	(0.7–1.3)	0.7900
Basiliximab	11,464 (77.2%)	1.2	(1.0–1.4)	0.0879
Rituximab	806 (5.4%)	0.8	(0.6–1.2)	0.2251
Initial calcineurin inhibitor				
Cyclosporine	3088 (20.8%)	1.0		
Tacrolimus	11,754 (79.2%)	1.0	(0.8–1.1)	0.8473
Use of mycophenolate mofetil	13,201 (88.9%)	1.0	(0.9–1.2)	0.7401
Initial steroid regimen				
Deflazacort	1605 (10.8%)	1.0		
Dexa/betamethasone	718 (4.8%)	1.3	(0.9–1.8)	0.1482
Fludro/hydrocortisone	1485 (10.0%)	1.4	(1.0–1.8)	0.0539
(Methyl)prednisolone	11,034 (74.3%)	**1.3**	**(1.0–1.6)**	**0.0402**
Acute rejection	1248 (8.4%)	1.0	(0.8–1.2)	0.7596

Abbreviations: HR, hazard ratio; CI, confidence interval; KT, kidney transplantation. Bold letters indicate statistically significant results. ^1^ Hospitals certified by the Ministry of Health and Welfare of Korea as specialized in high-level medical practices.

## Data Availability

Restrictions apply to the availability of these data. Data were obtained from the National Health Insurance claim for all Korean residents, are administered by the Korean National Health Insurance Service, and are available from the National Health Insurance Sharing Service (https://nhiss.nhis.or.kr/, accessed on 13 March 2021) with the permission of the National Health Insurance claim.

## Supplementary Materials

The following are available online at https://www.mdpi.com/article/10.3390/jcm10163530/s1, Table S1: No. of kidney transplant recipients according to age and sex groups in this study, Table S2: No. of kidney transplant recipients by sex and years of KT surgery in this study, Table S3: De novo primary cancer incidence during the follow-up period after kidney transplantation in a total of 14,842 patients, Table S4: Types of post-kidney-transplantation de novo cancers, including different types occurring in the same patient.
