# Supplementary material for: De Novo Cancer Incidence after Kidney Transplantation in South Korea from 2002 to 2017
Source: J Clin Med. 2021 Aug 11;10(16):3530. doi: 10.3390/jcm10163530 (PMC8396914; doi:10.3390/jcm10163530)
Supplement: Supplementary file 1 [file jcm-10-03530-s001.zip › jcm-1316624-supplementary.pdf]

Table S1. No. of the kidney transplant recipients according to the age and sex groups in this study.

Age at KT (years)	No. of kidney transplant recipients		
	Male	Female	Total
0 - 9	45	37	82
10 - 19	195	116	311
20 - 29	646	440	1,086
30 - 39	1,626	1,236	2,862
40 - 49	2,363	1,705	4,068
50 - 59	2,651	1,846	4,497
60 - 69	1,106	690	1,796
≥70	97	43	140
Total	8,729	6,113	14,842

Abbreviations: KT, Kidney transplantation; No, Number.

Table S2. No. of the kidney transplant recipients by the sex and years of KT surgery in this study.

Year	No. of kidney transplant recipients		
	Male	Female	Total
2002	288	164	452
2003	313	202	515
2004	274	220	494
2005	247	191	438
2006	323	262	585
2007	403	288	691
2008	473	400	873
2009	530	431	961
2010	559	398	957
2011	677	450	1,127
2012	721	503	1,224
2013	692	487	1,179
2014	696	488	1,184
2015	745	471	1,216
2016	896	596	1,492
2017	892	562	1,454
Total	8,729	6,113	14,842

Abbreviations: KT, Kidney transplantation; No, Number

Table S3. De novo primary cancer incidence during the follow-up period after kidney transplantation in a total of 14,842 patients.

Year	No. of KT patients diagnosed with any de novo cancer			No. of KT patients (Death-censored, accumulated)			Annual cancer incidence of this study[1]			NCIC annual cancer incidence of South Korea[2]		
	Male	Female	Total	Male	Female	Total	Male	Female	Total	Male	Female	Total
2002	5	2	7	283	161	444	1766.8	1242.2	1576.6	N/A	N/A	N/A
2003	3	6	9	588	361	949	510.2	1662.0	948.4	N/A	N/A	N/A
2004	5	3	8	853	576	1,429	586.2	520.8	559.8	N/A	N/A	N/A
2005	5	7	12	1,095	762	1,857	456.6	918.6	646.2	N/A	N/A	N/A
2006	12	16	28	1,404	1,011	2,415	854.7	1582.6	1159.4	338.6	295.0	316.8
2007	9	16	25	1,792	1,286	3,078	502.2	1244.2	812.2	N/A	N/A	N/A
2008	11	15	26	2,247	1,672	3,919	489.5	897.1	663.4	383.7	356.0	369.9
2009	13	18	31	2,757	2,082	4,839	471.5	864.6	640.6	N/A	N/A	N/A
2010	35	21	56	3,284	2,456	5,740	1065.8	855.0	975.6	424.2	409.7	417.0
2011	27	40	67	3,924	2,888	6,812	688.1	1385.0	983.6	447.4	439.3	443.4
2012	49	35	84	4,591	3,366	7,957	1067.3	1039.8	1055.7	454.3	453.0	453.6
2013	70	30	100	5,225	3,823	9,048	1339.7	784.7	1105.2	457.5	449.2	453.4
2014	76	56	132	5,858	4,278	10,136	1297.4	1309.0	1302.3	452.5	417.2	434.8
2015	76	58	134	6,526	4,701	11,227	1164.6	1233.8	1193.6	451.4	403.7	427.6
2016	103	71	174	7,349	5,253	12,602	1401.6	1351.6	1380.7	476.5	432.0	454.2
2017	106	51	157	8,142	5,770	13,912	1301.9	883.9	1128.5	484.9	434.7	459.8
Total	605	445	1,050	8,142	5,770	13,912						

Abbreviations: KT, kidney transplantation; NCIC, National Cancer Information Center; N/A, not available; [1] annual cancer incidence per 100,000 persons; [2] Data from NCIC annual cancer incidence of South Korea for the years 2002 to 2005, 2007, and 2009 were not publicly available..

Table S4. Types of post-kidney transplantation de novo cancers, including different types occurring in the same patient.

Site of cancer	Integrated classification	ICD-10 code	Male (Death-censored, N = 8,142)		Female (Death-censored, N = 5,770)		Total (Death-censored, N = 13,912)	
			N	Incidence (%)	N	Incidence (%)	N	Incidence (%)
Tongue base	Tongue	C01	1	0.012			1	0.007
Other and unspecified parts of tongue	Tongue	C02	1	0.012	1	0.017	2	0.014
Gum	Mouth	C03	1	0.012			1	0.007
Mouth floor	Mouth	C04			1	0.017	1	0.007
Other and unspecified parts of mouth	Mouth	C06	3	0.037	1	0.017	4	0.029
Parotid gland	Salivary glands	C07	2	0.025	1	0.017	3	0.022
Tonsil	Tonsil	C09	3	0.037			3	0.022
Oropharynx	Oropharynx	C10	1	0.012			1	0.007
Nasopharynx	Nasopharynx	C11	3	0.037			3	0.022
Esophagus	Esophagus	C15	6	0.074	2	0.035	8	0.058
Stomach	Stomach	C16	62	0.761	33	0.572	95	0.683
Small intestine	Small intestine	C17	1	0.012			1	0.007
Colon	Colon	C18	34	0.418	30	0.520	64	0.460
Rectosigmoid junction	Rectum	C19	9	0.111	11	0.191	20	0.144
Rectum	Rectum	C20	10	0.123	6	0.104	16	0.115
Anus and anal canal	Anus	C21	1	0.012	2	0.035	3	0.022
Liver and intrahepatic bile ducts	Liver	C22	83	1.019	43	0.745	126	0.906
Gallbladder	Gallbladder	C23	2	0.025	4	0.069	6	0.043
Other and unspecified parts of biliary tract	Gallbladder	C24	6	0.074	4	0.069	10	0.072
Pancreas	Pancreas	C25	26	0.319	14	0.243	40	0.288
Other and ill-defined digestive organs	Digestive tract	C26	1	0.012			1	0.007
Nasal cavity and middle ear	Nose, sinuses etc.	C30	2	0.025			2	0.014
Accessory sinuses	Nose, sinuses etc.	C31	2	0.025	1	0.017	3	0.022
Larynx	Larynx	C32	4	0.049			4	0.029

Bronchus and lung	Lung	C34	39	0.479	13	0.225	52	0.374
Thymus	Other thoracic organs	C37			1	0.017	1	0.007
Heart, mediastinum and pleura	Other thoracic organs	C38	2	0.025			2	0.014
Bone and articular cartilage of limbs	Bone	C40	1	0.012	1	0.017	2	0.014
Bone and articular cartilage of other and unspecified sites	Bone	C41	1	0.012	2	0.035	3	0.022
Malignant melanoma of skin	Malignant melanoma of skin	C43	2	0.025			2	0.014
Other malignant neoplasms of skin	Other skin	C44	34	0.418	16	0.277	50	0.359
Mesothelioma	Mesothelioma	C45	2	0.025			2	0.014
Kaposi's sarcoma	Kaposi's sarcoma	C46	11	0.135	4	0.069	15	0.108
Peripheral nerves and autonomic nervous system	Connective and soft tissue	C47	1	0.012			1	0.007
Other connective and soft tissue	Connective and soft tissue	C49	5	0.061	1	0.017	6	0.043
Breast	Breast	C50	2	0.025	61	1.057	63	0.453
Vulva	Vulva	C51			3	0.052	3	0.022
Vagina	Vagina	C52			1	0.017	1	0.007
Cervix uteri	Cervix uteri	C53			19	0.329	19	0.137
Corpus uteri	Corpus uteri	C54			10	0.173	10	0.072
Uterus, part unspecified	Uterus unspecified	C55			1	0.017	1	0.007
Ovary	Ovary	C56			25	0.433	25	0.180
Other and unspecified female genital organs	Other female genital organs	C57			1	0.017	1	0.007
Placenta	Placenta	C58			1	0.017	1	0.007
Penis	Penis	C60	1	0.012			1	0.007
Prostate	Prostate	C61	104	1.277			104	0.748
Testis	Testis	C62	2	0.025			2	0.014
Other and unspecified male genital organs	Other male genital organs	C63	1	0.012			1	0.007
Kidney, except renal pelvis	Kidney	C64	97	1.191	33	0.572	130	0.934
Renal pelvis	Renal pelvis	C65	5	0.061	6	0.104	11	0.079
Ureter	Ureter	C66	2	0.025	10	0.173	12	0.086
Bladder	Bladder	C67	21	0.258	14	0.243	35	0.252
Other and unspecified urinary organs	Other urinary organs	C68			1	0.017	1	0.007
Eye and adnexa	Eye	C69	1	0.012			1	0.007
Brain	Brain	C71	4	0.049	5	0.087	9	0.065
Thyroid gland	Thyroid	C73	49	0.602	105	1.820	154	1.107
Other endocrine glands and related structures	Other endocrine	C75	2	0.025	1	0.017	3	0.022
Other and ill-defined sites	Other	C76	2	0.025			2	0.014
Secondary and unspecified malignant neoplasm of lymph nodes	Lymph nodes metastasis	C77	22	0.270	29	0.503	51	0.367
Secondary malignant neoplasm of respiratory and digestive organs	Metastasis	C78	36	0.442	25	0.433	61	0.438
Secondary malignant neoplasm of other and unspecified sites	Metastasis	C79	26	0.319	24	0.416	50	0.359
Malignant neoplasm without specification of site	Disseminated malignant neoplasm	C80	5	0.061	4	0.069	9	0.065
Hodgkin lymphoma	Hodgkin lymphoma	C81	1	0.012			1	0.007
Follicular lymphoma	Non-Hodgkin lymphoma	C82	1	0.012			1	0.007
Non-follicular lymphoma	Non-Hodgkin lymphoma	C83	33	0.405	22	0.381	55	0.395

Mature T/NK-cell lymphoma	Non-Hodgkin lymphoma	C84	3	0.037			3	0.022
Other and unspecified types of non-Hodgkin lymphoma	Non-Hodgkin lymphoma	C85	23	0.282	15	0.260	38	0.273
Other specified types of T/NK-cell lymphoma	Non-Hodgkin lymphoma	C86	2	0.025			2	0.014
Malignant immunoproliferative diseases	Immunoproliferative diseases	C88	3	0.037	7	0.121	10	0.072
Multiple myeloma	Multiple myeloma	C90	4	0.049	4	0.069	8	0.058
Lymphoid leukemia	Lymphoid leukemia	C91	2	0.025	1	0.017	3	0.022
Myeloid leukemia	Myeloid leukemia	C92	6	0.074	3	0.052	9	0.065
Other leukemias of specified cell type	Myeloid leukemia	C94	1	0.012	1	0.017	2	0.014
Leukemia of unspecified cell type	Leukemia unspecified	C95			1	0.017	1	0.007
Lymphoid, hematopoietic and related tissue, other and unspecified	Non-Hodgkin lymphoma	C96	2	0.025			2	0.014
Total			825	10.133	625	10.832	1,450	10.423

Abbreviations: ICD-10, International Statistical Classification of Diseases and Related Health Problems 10th Revision.
